# Formality or Reality: Student Teachers’ Experiences of Ethical Dilemmas and Emotions During the Practicum

**DOI:** 10.3389/fpsyg.2022.870069

**Published:** 2022-06-02

**Authors:** Xin Wang, Denghui Liu, Jingyan Liu

**Affiliations:** School of Humanities, Jiangnan University, Wuxi, China

**Keywords:** teacher student, ethical dilemma, emotion experience, qualitative research, teacher education, practicum

## Abstract

The main purpose of the study was to explore Chinese student teachers’ experience during their practicum and what they reported as ethical dilemmas and how these experiences affected them in terms of professional development as well as emotional well-being. Through the analysis of qualitative data collected from forty-three participants’ interviews, this study identified six most commonly reported ethical dilemmas, including: formal curriculum versus informal and hidden curriculum; family agenda versus educational standards; loyalty to colleagues versus school norms; confidentiality versus school rules; conformism dilemmas and red-envelope dilemmas and found that dilemmas about formal curriculum versus informal and hidden curriculum were the dominant workplace ethical dilemma for student teachers. The results also showed that the majority of the student teachers reported experiencing negative emotions or painful memories, which may hinder their development of professional competencies and overall wellbeing. Thus, the study argues that student teachers’ frequent encounters with ethical dilemmas highlight the challenges faced by teacher educators in transferring knowledge between university and school environments, and student teachers should be equipped with essential emotional regulation strategies that could benefit them in their future work.

## Introduction

Ethical dilemmas refer to conflictual ethical situations that require undesirable choices between competing values and expectations that cannot be simultaneously and fully satisfied (Cuban, 2001). An ethical dilemma can be characterized by the simple question “What ought I to do?” in a given situation.

According to the literature, some regulated professions, such as teaching, nursing, and medicine, are more likely than other professions to encounter ethical dilemmas in reality, as they play an important role in providing essential services to the public on a daily basis (Lyons, 1990; Yin, 2015; Lilach, 2020). Commonly confronted ethical dilemmas for teachers may involve dealing with issues such as irresponsible colleagues, pupils who have been treated unfairly, and inappropriate discussion about the students/staff. When their understanding of proper behavior is restricted by complex factors in reality and decisions are made or actions are carried out contrary to the “right way”, ethical dilemmas and moral distress arise (Shapira-Lishchinsky, 2011), which may have a negative impact on their emotions and professional competencies (e.g., experiences of emotional distress, feeling inadequate and unintelligent) (D’eon et al., 2007; Wang and Ho, 2020).

While there is a growing body of literature focused on in-service teachers’ experiences of ethical dilemmas (Campbell, 1997, 2006; Husu and Tirri, 2001; Aultman et al., 2009; Ehrich et al., 2011; Shapira-Lishchinsky, 2011), student teachers’ experiences of ethical dilemmas and their emotional experiences have received less attention. This study aims to bridge this gap by uncovering ethical dilemmas encountered by student teachers during the practicum, and how they are emotionally affected by such experiences.

## Background

Previous studies have examined in-service teachers’ workplace-based ethical dilemmas at various levels, including kindergarten (e.g., Husu and Tirri, 2001; Tirri and Husu, 2002), primary school (Tirri and Husu, 2002; Ehrich et al., 2011), secondary school (Tirri, 1999; Campbell, 2003; Ehrich et al., 2011), and college and university (Helton and Ray, 2005).

However, student teachers’ experience of ethical dilemmas received less attention than required. To promote better teacher education, the scholarly body has focused on student teachers’ formal education in terms of knowledge development and belief formation, and a rather fundamental fact of teacher education has been unintentionally ignored: not all of what is taught during teacher training is captured in formal curricula such as course catalogs, lecture notes and handouts. In fact, a great deal of what is taught and most of what is learned takes place outside of the classroom, as learning is also a cultural process that is affected by a number of structural factors, such as customs, rituals, and taken-for-granted attitudes and behaviors, and many other factors that do not exist in formal classes (Hafferty, 1998).

These external forces sometimes play a vital role in constructing commonly held “understating” and “beliefs” about what is good and bad education, what is valued within the community, and what are the accepted behavior norms for the profession (Hafferty, 1998; Karnieli-Miller et al., 2010). According to occupational socialization theory, when there is a gap between these external forces and the students’ beliefs that they have developed during formal education, “reality shock” is likely to happen, and this is one of the main reasons described by new teachers as to why they leave the profession (Ingersoll and Smith, 2004). Student teachers’ beliefs play an important role in terms of understanding their experiences of ethical dilemmas during practicum, as they form part of the process of understanding how student teachers conceptualize their teaching practices which in turn is important to the understanding of their reac-tions and decisions when confronted ethical dilemmas. A growing body of research argues that the relationship between teachers’ beliefs and practices is intertwined and context de-pendent (Mansour, 2009), therefore teacher students’ beliefs and their experiences of ethical dilemmas should be examined together within the context of practicum.

Despite the “reality shock,” many of the ethical dilemmas can also be attributed to the lack of adequate practicum supervision. According to the literature, some teaching practicums are still largely based on old, exclusive paradigms of knowledge production that is disconnected from context and new epistemology creates expanded learning opportunities for stu-dent teachers and better prepares them for successful interaction in complex teaching prac-tices is needed (Zeichner, 2010). Also, Chinese and other higher education contexts are characterized by government funding reduction, high staff–student ratios may present chal-lenges in finding appropriate supervising teachers for student teachers during their practicum too (Mabunda, 2013).

Meanwhile, according to the theory of emotion, students’ experience and emotion are not independent. Their emotions depend on their experiences, and different patterns of experiences may create different emotions (Stangor and Walinga, 2014). There are a number of researchers investigating students’ experiences of ethical and professionalism dilemmas, and their research results indicated that students’ emotional experiences in responding to ethical dilemmas can affect their development of professional competence and their occupational well-being in the future (Wang and Ho, 2020, 2021). According to the literature, there are three levels of emotions that are key to teachers, including primary emotions and social emotions (Damasio, 2004). Primary emotions include fear, anxiety, anger, disgust, surprise, sadness and happiness, which are likely to be present in teachers’ work. If negative primary emotions persist, they may result in the loss of a sense of wellbeing by teachers in their ability to perform professionally. Social emotions, including “sympathy, embarrassment, shame, guilt, pride, jealousy, envy, gratitude, admiration, indignation and contempt” (Damasio, 2004), are more context-related. For example, teachers may experience shame and guilt when they feel that they have failed to meet their own or others’ moral standards (Hargreaves, 1998). Pupils have also spoken of sympathy and praise (related to gratitude and admiration) as being associated with “good” teachers and embarrassment, shame and contempt as being associated with “bad” teachers.

Similar to teachers, student teachers’ capacities are highly likely to be affected by their emotional experiences during the practicum, as they carry out tasks similar to those of teachers and are treated as teachers-in-practice by pupils and other professionals in the field. While researchers have investigated teachers’ emotions in a variety of educational contexts and highlighted how emotions are inextricably linked to teachers’ work, development and identity (e.g., Hargreaves, 2005) and how those emotions affect teachers’ lives and well-being (e.g., Zembylas, 2005), to date, there has been no significant effort made to examine student teachers’ emotional experiences during and after confronting ethical dilemmas in the practicum. The few papers that have studied student teachers’ dilemmas either did not qualitatively examine their emotional experiences in responding to these dilemmas (Donahue, 1999; Meijer et al., 2011; Shapira-Lishchinsky, 2011; Lilach, 2020) or only carried out a case study with a very small sample size that failed to reveal the bigger picture (Deng et al., 2018).

## The Aim of the Study

In an effort to add one thread of evidence to the larger fabric of information that needs to be woven, we carried out the current study with a systematic qualitative analysis of student teachers’ experience during their practicum and what they reported as ethical dilemmas and how these experiences affected them in terms of professional development (e.g., whether they taught them something about teaching as a profession and professional values) as well as emotional well-being (e.g., feelings of anxiety, stress, or depression). Analyzing how ethical dilemmas have been dealt with, such as how certain activities are accepted, encouraged, or undermined by the institution, the policy or the “group talk”, also helps to “open a window” on the informal and hidden curricula on which the current literature is mostly theoretical and not grounded in empirical evidence from measures of students’ experiences (Karnieli-Miller et al., 2010), which could help to understand how student teachers’ professional development has been shaped by the hidden and informal curriculum, because ethical dilemmas are one of its most important constituents.

In summary, the present study was designed to address the following research questions:

(1) Do student teachers experience ethical dilemma(s) during their practicum?
(2) What type of ethical dilemmas do student teachers experience?
(3) What impact (if any) do student teachers perceive emotionally?

## Materials and Methods

### Context of the Study

Teacher education in China is composed of two parts: preservice education and in-service training. Preservice education includes (1) a four-year teacher training program provided by normal universities and comprehensive universities with teacher education programs; (2) three-year teacher training colleges; and (3) secondary teacher training schools. These institutions train teachers for senior and junior secondary schools, primary schools, and kindergartens, respectively. In-service training is conducted by education institutes and in-service teacher training schools for teachers from different levels.

Teacher education programs provided by universities are typically 48 months of duration and include both theoretical and experiential components. Formal education courses taken at universities provide the theoretical foundations and teaching tools, while the school practicum provides the opportunity to put these theories and ideas into practice.

The program under study is a four-year program provided by Jiangnan University, a comprehensive university in Jiangsu Province, eastern China. As a national key “211 Project” university, it is one of the largest universities in the province, with over 32,219 students and 3,430 staff in total. Teacher education is one of the major programs offered in its education department, with over 480 students enrolled in 2021. This program provides two directions for students to choose: one direction focuses on Chinese language and literature teaching, and the other focuses on mathematics teaching.

According to the regulation of the department, student teachers need to complete a 4-week practicum in Year 3 and another 12-week practicum in Year 4 before they can obtain the credit required for graduation. The supervision of student teachers during the program is shared between faculty advisers (university faculty members) and school advisers (usually experienced school teachers). In the program, faculty members teach courses and are assigned to a group of student teachers (usually 3-6) whom they need to supervise throughout the program, including the practicum. However, the main supervisor during the practicum is the school advisor.

In total, the education department has 15 partner schools that function as the “assigned practicum school group”. The program aims to send student teachers to the same practicum school for his or her entire 16-week practicum (4 weeks in year 3 and 12 weeks in year 4) in order to create a support system on site. In theory, the faculty advisor and the school advisor observe student teachers during the practicum; the school advisor is the director supervisor who spends time with the student every day providing support, advice, guidance and feedback when needed. The faculty advisors visit the school and observe student teachers every week, depending on their own schedule.

Each student is required to complete a practicum diary to record their “journey of learning”. For year 3 students, their diary mainly contains course evaluations and personal reflections, while for year 4 students, as they have moved to the more advanced part of the practicum in which they are required to deliver 2-3 classes based on their major, their diary also includes reflections on his or her own teaching practices. At the end of the practicum, the faculty advisors and the school advisors both go over an evaluation checklist and make a joint decision on the outcome of the practicum. In cases of disagreement, the decision is passed to the practicum coordinators and the head of the department.

### Research Design

This study is part of a larger research project investigating Chinese student teachers’ narratives of ethical dilemmas. Narrative inquiry is used to explore and make sense of student teachers’ experience of ethical dilemmas (Connelly and Clandinin, 1990; Clandinin, 2006). Narrative is a widely used qualitative research method that is grounded in interpretive hermeneutics and phenomenology and provides “insight that befits the complexity of human lives” by gathering narratives and analyzing the meaning that individuals ascribe to their experiences. It can highlight ethical matters and shape new interpretations of people’s experiences and is often used in studies of lived experience (Wang and Ho, 2020). We used narrative interviews and focus groups as our main data collection methods to gather students’ lived experiences of ethical dilemmas.

### Participants

We invited year 3 and year 4 student teachers from the education department to participate through announcements by class representatives and department administrators, online notice boards, and through snowballing. We limited our recruitment to the senior years of undergraduates because they have practicum experience and, as such, are more likely to have encountered ethical dilemmas. Following ethical approval, we recruited 43 students to participate in this study, including 22 students in Year 3 and 21 students in their final year of study. The number of participants from each study group was as follows: Chinese language and literature teaching (n = 20) and mathematics teaching (n = 23). The male to female gender ratio in our study sample was very close to that of the overall student population (see Table 1). We stopped recruiting new participants when the interviews no longer provided new information and the themes stabilized to reach “sample saturation”.

**TABLE 1:** Study participant demographics.

Study Subject	Number of Participants	Average Age	Male	Female	Gender ratio (sample)	Gender ratio (total)*
Chinese language teaching	20	21	2	18	1:9	1:8.1
Mathematics teaching	23	21	3	20	1:6.7	1:7.2

*This refers to the male to female gender ratio for the total number of students in the corresponding years of study.

### Data Collection

We grouped students by study interest and year and then conducted eight focus group interviews with a total of 43 students (38 females, 5 males). Each focus group has a researcher as interviewer and 3 to 8 student interviewees. Participation was confidential and voluntary. All participants provided their written informed consent to participate in this study. We developed a group interview guide based on previous literature to ensure consistency in interviewing across all groups. Due to the different availabilities of participants, the interviews were scheduled across 6 days. All three researchers took on the interviewing task (XW interviewed 4 groups, LDH interviewed 2 groups, and LJY interviewed 2 groups). After a general welcome and a warm-up free talk on their experience of practicum, we introduced the ground rules and started each group interview with a general question to uncover student teachers’ understanding of professionalism and ethical dilemmas. They were then asked to describe an example of ethical dilemmas that they had experienced or witnessed and to reflect on the experience. Students were encouraged to speak freely and openly and to include what they did, why they did it and how they felt about it. All interviews were conducted in Mandarin and audio recorded with permission from participants. Respective quotes have been translated into English and are presented in the Results section.

### Data Analysis

The recorded interviews were transcribed and anonymized for analysis, which was facilitated by NVivo 12 software (QSR International Pty Ltd., Doncaster, VIC, Australia). We initially coded the transcripts using template analysis (King, 2004) because it is flexible in terms of using predefined themes and allows the researcher to make adjustments (such as adding new codes or deleting irrelevant ones) during the coding process (King, 2004; Slade et al., 2009).

We adopted a coding framework developed by Shapira-Lishchinsky (2011) as *a priori* template for our analysis. In the first stage, all three researchers read and coded the same copy of the interview transcript individually according to the *priori* template, using NVivo12. In the second stage, researchers revisited the data and reviewed the coding together to resolve disagreement through group meetings as well as to set-up group working rules to ensure consistency in coding across all transcripts. In the third stage, two researchers (Wang and Liu JY) read and coded the rest of the transcripts individually according to the *priori* template. Finally, all three researchers sat together to cross-check the coding and resolve disagreements through discussion. As a result, two themes that were not found in our data were removed from the original template, and three new themes were added, including red-envelope dilemmas, formal versus informal and hidden curriculum dilemmas, and conformism dilemmas. Our final coding template includes six themes and will be presented in the Results section (see Table 2 for the full list).

**TABLE 2:** List of themes.

1. Dilemmas about formal curriculum versus informal and hidden curriculum 2. Dilemmas about family agenda versus educational standards 3. Conformism dilemmas
4. Red-envelope dilemmas 5. Dilemmas about loyalty to colleagues versus school norms
6. Dilemmas about confidentiality versus school rules

## Results and Analysis

### Types of Dilemmas

**FIGURE 1:**
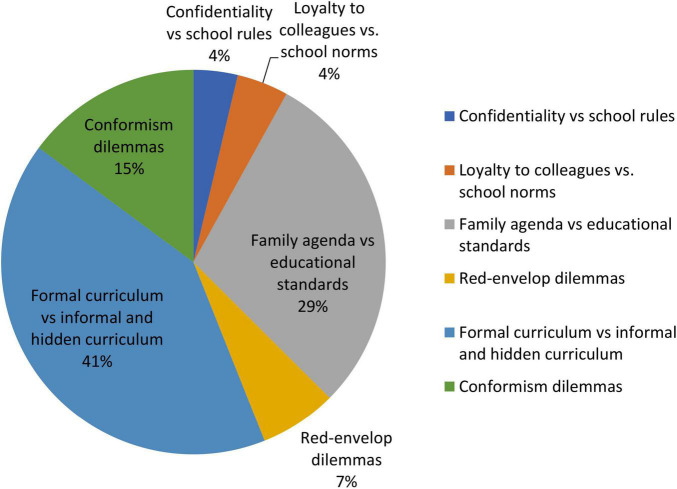
Distribution of ethical dilemmas.

Of the 43 interviewed students, 326 personal incident narratives (PINs) were identified and coded to one or more than one of the six themes (see Table 2). Dilemmas about formal versus informal and hidden curricula were the most commonly reported (*n* = 133), followed by family agenda versus educational standards dilemmas (*n* = 95), conformism dilemmas (*n* = 48), red-envelope dilemmas (*n* = 21), dilemmas about loyalty to colleagues versus school norms (*n* = 14), and dilemmas about confidentiality versus school rules (*n* = 12) (see Figure 1 for the distribution of dilemmas).

#### Dilemmas About Formal Curriculum Versus Informal and Hidden Curriculum

This is the category most frequently discussed by student teachers during their practicum. Of the 326 identified PINs, 133 were coded to this theme. This theme focuses on student teachers’ ethical dilemmas caused by the disconnection between their formal curriculum and the informal and hidden curriculum. Not all of what is taught during teacher training is captured in formal curricula (such as course catalogs, lecture notes and handouts), but within what is called the “hidden curriculum”, which mainly consists of students’ experiences of and responses to the real-world working environment (Hafferty, 1998).

The informal and hidden curriculum stands in direct contrast to the formal curriculum in terms of their primary targets. Unlike the formal curriculum, the informal and hidden curriculum addresses the importance of role models and sees education as a process that is affected by a number of structural factors, such as customs, rituals, and taken-for-granted attitudes and behaviors, and many other factors that do not exist in formal classes (Hafferty, 1998).

The following three examples demonstrated students’ experience of the dilemma between the formal and informal and hidden curriculum in terms of “the accepted behavior norms” for teachers. The first example is one of the Year 3 students who reported that he witnessed a student being treated inappropriately, which gave him a huge “reality shock”:

*I remember in one Chinese class, there was a boy called Tian who forgot to do his homework. His Chinese teacher literally threw the exercise book at him and asked him to pick it up, hand it back, and threw it at him again. I was totally shocked. I mean, that was definitely not okay according to what we were taught in the university. (Year 3 Student 2, Male, Group 1)*

In this incident, the student was shocked to witness such behavior in the first place. In addition, he was then confused by others’ reactions because apart from him, nobody seemed to care. The student explained that, in theory, the teacher’s behavior was against the rules; however, in practice, everyone else treated this kind of behavior as normal. The student confessed that he was feeling confused about which “behavior code” he should follow.

Other students also reported cases in which children were humiliated by their teachers in front of other students or in public. In the second example, a Year 3 student reported witnessing an incident in which a girl was verbally abused by her teacher:

*That girl was not doing well in her math. In fact, her academic performance was well below average. And in their last math test, she got something like*… *14 out of 100. The math teacher was very angry with her and asked her to come to the front and said, “You need to ask your mum and dad to take you for an IQ test, seriously. I am not sure if you are normal for your age group*…*” right in front of the whole class. (Year 3 Student 7, Female, Group 4)*

This example again demonstrates the student teachers’ experience of colleagues’ inappropriate behavior. In this case, the math teacher used humiliating language in front of the class to insult the girl who failed the math test. After the incident, the student teacher was not just confused but also sad, angry and regretful for not being able to stand up to the math teacher. She now believes that this kind of incident should not be tolerated, even though it would mean facing the teacher who has more power over her.

In the third example, one of the student teachers reported hearing another teacher talking about a boy’s father’s career in a quite disrespectful way:

*In the other class, there was a boy named D, and one afternoon, he came to the teachers’ office to submit his homework, the teacher started a conversation and asked “What does your dad do?” The boy said his dad works at the funeral parlor. And then, the teacher asked if he had been there before. The student said yes. The teacher then asked, “Are you scared?” The boy said no. The teacher then had a shocked expression and said in a really high pitch, “What? You are not scared? How come? Are you also a dead man?” (Year 4 student 5, Female, Group 7)*

In this incident, although the student teacher was sure that the teacher’s language and behavior was not acceptable according to the rules, she decided to remain silent because she felt “powerless” to stop the teacher and felt deeply sorry for not being able to help the little boy out.

These three narratives reveal the fact that student teachers reported experiencing a large volume of ethical dilemmas related to “reality shock” in the early stages of their practicum. According to occupational socialization theory, this is mainly caused by a disconnection be-tween what was honored by their formal curriculum and what was practiced in reality. It can be learned from the narratives presented above that, on experiencing these dilemmas, students find significant discrepancies between what they envisioned as student teachers and what they are actually experiencing during their practices of teaching. And this gap between their formal and hidden curriculum is the main reason contributing to the development of most reported ethical dilemmas in our study.

#### Dilemmas About Family Agendas Versus Educational Standards

This is the second most commonly reported dilemma. It refers to the question of whether teachers should compromise their professional standards to meet a student’s family’s request. The first example describes a clash between a teacher’s desire to make a professional decision in terms of developing the social skills of the pupil and the pupil’s family’s request with which the teacher does not agree. In this incident, the teacher did not bend under pressure.

*This happened about 2 weeks ago. There was a girl from grade 2, and she had a little conflict with her desk-mate and she cried. Her mother found out about it the next day and asked us to step in and punish the boy for making her daughter cry. The teacher (who is my school tutor) explained that it was just a very small conflict that happened between children, and it was a good learning opportunity for the girl to develop her social skills, and the teacher was ready to help her. But the mum insisted that the teacher should take full responsibility for everything that happened in school, rather than leaving students to solve problems on their own. (Year 4, student 8, Female, Group 4)*

In the second example, the student teacher went for a home visit with his school tutor and experienced tension between academic standards and the parent’s expectations.

*I went for a home-visit with my school tutor the other day, and it was a quite*… *unexpected experience. This boy had some problems with his math and Chinese literature, which are the main courses for his grade. So we decided to visit his parents first and schedule more time for him. But just as we started to talk about the boy’s problems in terms of his academic performance, his parents said something like “We are sorry teacher, but we are aiming for a happy education for our son, and we don’t really see academic achievement as a big deal, you know. We are a wealthy family; we just want our son to be happy.” And then both of us were silent. You know, there is no way you can grab their attention by talking about their son’s learning problems in math or Chinese, as they already make it clear that they don’t care about that at all. So, we had a little talk about something else and said goodbye. (Year 3, student 6, Male, Group 4)*

After the visit, the student teacher reflected on his experience and said he was feeling sad and powerless when faced with this dilemma. His response is that next time, he will not let parents compromise his educational standards. He will make clear to the parents what the reasonable academic expectations of the pupil are, as well as the academic requirements of the school, and expect the pupil’s parents to respect these standards.

The third example is a similar situation in which the pupil’s parents took the girl for a fashion show performance, causing the pupil to miss many classes. In this example, the teacher’s response was to ask for help from the school headmaster.

*Well, I was there with the math teacher in his class, and the girl’s mum came in to take her daughter out for a fashion show competition. And she explained that she wants her daughter to be a super model, and school is not important for her at all. (Year 3, student 1, Female, Group 2)*

While in the previous example, the teacher escaped the conversation without clarification or explanation, in this example, in contrast, the math teacher decided to ask for wider support in order to stand by his professional values. This course of action, according to the student teacher, made the dilemma easier to handle and caused less depressed feelings for the student teacher.

Student teachers reported experiencing this type of ethical dilemma mainly because they did not know how to deal with conflicts between family agendas and educational standards. This can be seen as generally attributed to the lack of adequate practicum supervision. As mentioned before, most the teacher trainings programs are still based on old, exclusive paradigms of knowledge production that are disconnected from real context; diverse learning opportunities that help student teachers to develop transferable skills that are needed in complex teaching practices, such as interpersonal relationship skills, conflict management skills, effective communication skills are not adequately provided by their training institutions. And this “lack of adequate practicum supervision” is the main reason contributing to the development of the second most reported ethical dilemmas in this study.

#### Conformism Dilemmas

Here, the focus is on student teachers’ experiences of tension caused by the “mismatch” between themselves and their school advisors. Because the latter group plays an important role in student teachers’ successful completion of the practicum, many student teachers admitted that conformism is not only important but also necessary. In the first example, the student shared her experiences of being micromanaged by her school advisor, who expected the student teacher to copy his style instead of exploring her own.

*It feels like I don’t have my own experiential learning at all. I had to follow his lesson plan, his teaching style, and his ways of communicating with the pupils. He expects me to follow exactly what he asked, so I don’t mess things up or go in the wrong direction. But, I mean, I would like to have the freedom to do things my own way and learn from it. I felt like I was kept in this little box, and he wanted me to copy everything he did (Year 4, Student 6, Female, Group 5).*

Another interviewee shared how he was “muted” by his school advisor when he tried to discuss a certain professional issue:

*I was placed with a school advisor who has a strong personality. And in one of his math classes, he showed the student how to do the calculation, but it was not the best way to do it because it was more complicated than necessary for grade 4 students, and there is a simple and more straightforward way to do it. I tried to talk to him about it, and he simply said, “Thank you, but I think students would learn better using my way.” Although I strongly disagreed with him, I didn’t want to challenge his authority, so I kept my mouth shut. You know, I need him to sign my practicum evaluation form at the end of the practicum anyway (Year 4, Student 7, Male, Group 6).*

These examples reflected the fact that school advisors tend to take the role of “role modelers” for student teachers and typically expect them to model their practice after their own. However, by copying the school advisors’ styles, student teachers have been limited in terms of exploring their own teaching style as well as enacting their agency. Many students felt that they had to compromise to please their school advisors to get a passing mark on their practicum evaluation form, even when it meant they had to prioritize the school advisor’s will or value over their own. According to the students, this kind of compromise often made them feel depressed and unimportant.

Again, this is also attributed to the lack of adequate practicum supervision as mentioned before in the previous section. Due to a lack of training in transferable skills such as effective communication skills and interpersonal relationship management skills, some student teachers may feel helpless and vulnerable while interacting with their school advisor who has more power than themselves, especially when their relationship was less fortunate. And this is the main reason contributing to the development of the third most reported ethical dilemmas in our study.

#### Red-Envelope Dilemma

This dilemma refers to cases in which pupils’ parents buy gifts or vouchers (typically enclosed in a red envelope) for the teacher in exchange for special care or VIP tutorial sessions for the pupil. In most of the reported cases, the involved teachers refused to accept the gift/voucher and reclaimed their professional stands. In rare cases, teachers were reported to accept these gifts and vouchers. One student teacher reported witnessing a teacher in her office accepting expensive skincare sets from a girl’s mother:

*Yeah, I know of something similar. There was a girl whose mother bought a whole set of Estee Lauder skincare products for some of her teachers. I can’t say her name. Oh, yeah, a whole set, and it was wrapped in plastic shopping bags. I am not sure if the girl received any VIP tutorial sessions after that, but I don’t think it was appropriate for the teacher to accept such expensive gifts from the parents (Year 3, Student 3, Female, Group 3).*

In the second example, a student teacher reported witnessing a teacher returning a red envelope that contained gift cards and vouchers to the sender:

*I remember that was a Chinese teacher who had a private chat with a boy’s mum in her office, and I am not sure how she managed to do it, but the teacher found out that the boy’s mum left a red envelope on her desk, placed under some books. And he said he ran out straight away to catch up the mum and returned it. There were about 2 or 3 gift cards, I am not sure about the value. The teacher returned it immediately, and I think he did the right thing (Year 3, Student 7, Female, Group 2).*

These two transcripts reflect the fact that, as discussed before, teaching and learning is also a cultural process that is affected by a number of structural factors, such as customs, rituals, and many other factors that do not exist in formal classes (Hafferty, 1998). Therefore, the teacher training program should be grounded within the context of local culture and prepare students for the complex working environment.

#### Dilemmas About Loyalty to Colleagues Versus School Norms

Here, the focus is on the relationships between colleagues and pupils and the relationships among colleagues. The following two examples illustrate the topic. In the first example, the student teacher witnessed a teacher preparing for her “annual open class”, which means inspectors and professionals from outside come to her class for observation and provide a formal evaluation.

*And the teacher got really scared and started preparing for her open classes and other things. She told me that this open class would be “an act”, just to please these inspectors and get her good feedback. She had a rehearsal of the class, and it’s far from what I would call an original class. And I think it was a waste of time for the inspector and the external professionals because it is not what they expected to see (Year 4, Student 2, Female, Group 5).*

Although the student teacher believed that it was not the right thing to do, she also showed understanding for the teacher and therefore experienced inner conflicts herself. The student’s response was that in the future, she would be more sensitive to the professional expectations of her, and she would still remain loyal to her colleagues.

In the second example, the student teacher witnessed a similar incident, but in this case, pupils were also involved in “cheating the outsiders”:

*I had a similar experience. For the inspection, this teacher said he was going to pick some smart kids and tell them all the right answer in advance. So, during the open class, these smart kids could lead the student discussion, and the class would go smoothly. And students who don’t behave will not be allowed to come. Well, I don’t think the students would benefit much from the class, you know, everything was arranged in advance. And the teacher should have served as a role model, preventing his students from cheating, not showing them how to do it. I think it was a sham (Year 4, Student 8, Female, Group 4).*

This narrative conveys a strong tension between remaining loyal to a colleague and the need to express her disagreement to her school advisor that the teacher’s behavior was inconsistent with basic norms and principles. The student believed that the right thing to do was to confront her advisor, but she felt the pressure of being loyal, and her advisor implied that she should not convey her feeling to other teachers. The student teacher’s response to this incident was that she was feeling quite “uneasy” with these behaviors but could only remain silent.

These transcripts also relate to the “reality shock” in the early stages of student teachers’ practicum. To be more specific, these two students in the above transcripts experienced some taken-for-granted attitudes and behaviors that don’t exist in their formal curriculum and found significant discrepancies between what they envisioned before in the university and what they are actually experiencing in reality.

#### Dilemmas About Confidentiality Versus School Rules

This theme depicts the dilemma between a teacher’s desire to be discreet and the obligation to obey school regulations. The teacher’s work often includes private and confidential issues. When pupils confided in a teacher, they were faced with a dilemma of whether to betray that trust or to break the regulation. In the following example, a student teacher encountered a confidentiality dilemma and decided to “obey school rules”.

*It happened during the last week of my practicum. That afternoon, I was playing a table game with some of the boys, and they shared a secret with me about a boy, who got a bit of a masturbation problem. The boys didn’t really understand what he was doing, but they saw it as something unusual. I felt that I might not have done the right thing, but I think I should let their class teacher know about this issue because there might be some other mental problems involved. It was a hard decision. I don’t want to feel that way again. (Year 4, student 4, Male, Group 8).*

This ethical dilemma caused the student teacher considerable emotional distress, and his reluctance to report the event because of the pupil’s trust conflicts with his obligation to obey school regulations. These regulations require that staff should report any potential risks pupils may have to prevent severe problems. In this narrative, the tension between the need to tell their class teacher and the pupil’s requirement keep it secret stems from his loyalty to school principles.

These transcripts reflect the fact that, as discussed before, students’ emotional experiences in responding to ethical dilemmas could affect their development of professional competence and their occupational well-being. Therefore, transferable skills training, such as how to regulate emotions, should also be provided in formal teacher training programs (Gong et al., 2013).

## Discussion

Previous studies of ethics and education have usually focused on teachers’ ethical dilemmas (Campbell, 1997, 2006; Husu and Tirri, 2001; Aultman et al., 2009; Ehrich et al., 2011; Shapira-Lishchinsky, 2011). The present research analyzed the less-studied group of student teachers’ experiences of ethical dilemmas and further explored their emotional experiences in responding to the dilemmas. In this study, we first collected ethical dilemmas that student teachers encountered or witnessed during their practicum. We then analyzed and categorized them, together with student teachers’ emotional responses to these dilemmas.

The findings of this study suggest that student teachers face ethical dilemmas in the course of their practicum. Six main categories of ethical dilemmas were identified: formal curriculum versus informal and hidden curriculum; family agenda versus educational standards; loyalty to colleagues versus school norms; confidentiality versus school rules; conformism dilemmas and red-envelope dilemmas.

The most frequent ethical dilemmas we found were those involving tensions between formal curriculum versus informal and hidden curriculum, indicating that student teachers were not well informed about the gap between formality and reality when they left the university for the real school environment. Ethical dilemmas were not explicitly discussed in their formal curricula; therefore, most of the students only had a very vague picture of critical incidents they might have to deal with in practice. When faced with ethical dilemmas, students experienced the “reality shock”. As Moore Johnson (2004) said, people familiar with schools know that stories about how teaching is an easy job are nothing but illusions. Good teaching is not only demanding but also exhausting work. This explains why some of the students experienced reality shock during their practicum. In reflection, interviewed students generally view themselves as “not well prepared for the challenge”, and the majority of the ethical dilemmas reported in this category stem from lack of confidence in knowledge and abilities due to lack of preparation for the situation and students failed to acknowledge the impact ethical dilemmas may have on themselves.

Another category of frequently-reported dilemmas worth exploring was tensions between family agendas and educational standards, which indicates that teachers may experience difficulties collaborating with parents during the time of educational reform when there are different sets of educational standards, especially when parents have their own interpretations of the new educational targets and requirements. Recently, China has initiated waves of educational and curricular reform to guide and instruct its schools to move from traditional exam-oriented teaching to “quality teaching” to deliver “holistic education” and provide the required human resources to support the development of a knowledge economy that suits its long-term developmental strategies. During the process of transformation, it is likely that some of the parents may apply different points of reference when they consider what is important for the children at the current stage and sometimes show disapproval of the teacher’s academic standards. Ethical dilemmas arise when the parents and teachers differ in their understanding of what the appropriate academic standards are for children, and it is up to teachers to critically evaluate and then decide where to stand firmly behind their academic decisions, adjust their decisions considering the parents requires, or to bend under pressure.

According to the results, it was found that ethical dilemmas are multifaced, and there is no single way to respond to similar dilemmas, which means that the same dilemmas may be interpreted and responded to differently by each individual. For example, in the category of family agenda versus educational standards, one student reported witnessing the teacher avoid conflicts with parents by escaping the discussion, while another student reported witnessing the teacher asking for help from the headmaster in order to protect their professional values and standards. These findings suggest that students should also be aware of different options that are available to them to deal with ethical dilemmas effectively in different circumstances.

Therefore, this study suggests that ethical guidelines that are general but widely applicable could be of great help for student teachers in terms of helping them deal with ethical dilemmas by providing tools and preventing partiality that may distort judgment. Although basic ethical rules for teachers are widely acknowledged for student teachers, due to the multifaceted nature of ethical dilemmas, critical thinking skills and reflection skills are more important for students to deal with ethical dilemmas effectively in reality, not blind compliance. Therefore, developing up-to-date guidelines through ethical education programs for student teachers based on their knowledge as well as their experiences can empower them to develop pluralistic attitudes and a complex understanding of moral dilemmas and to choose from available options with careful consideration.

The results also showed that the majority of the student teachers reported experiencing negative emotions such as a sense of uncertainty, regret, depression, feeling of inadequacy and unprofessionalism, and sometimes painful memories, even though they were trying to minimize their unpleasant feelings afterward. Due to the complex nature of teaching, in order to achieve and sustain a healthy state of wellbeing, student teachers need to know how to manage the cognitive and emotional challenges of working in sometimes difficult scenarios that vary according to life experiences and events, as well as how to develop and maintain effective interpersonal relationships with three groups of people: colleagues, pupils and parents. This is because, according to the literate, supportive colleagues, good teacher–pupil and teacher-parent relationships had a positive effect on teachers’ positive professional life and emotional well-being. When entering the profession, most of the student teachers have a sense of vocation and a passion to give their best to their pupils. For some, these could become eroded with the passage of time, and experiences of ethical dilemmas are unanticipated. According to the study, some student teachers might risk losing their sense of purpose and wellbeing that are so intimately connected with their professional identities, beliefs, and self-efficacy due to their strong negative emotional experiences after encountering significant ethical dilemmas.

Therefore, this study also suggests that psychology courses that focus on emotional regulation strategies and establishing effective interpersonal relationships should be provided for student teachers, using real-life scenarios as case study examples. By doing so, student teachers will be able to have a trial before entering the real school environment, be more confident when faced with ethical dilemmas, and be able to regulate their negative emotional experiences afterward.

## Conclusion

The field of ethics has been the focus of increasing attention in teaching; it is not surprising given that teaching is a moral activity that is heavily value-laden (Ehrich et al., 2011). Student teachers’ experiences of ethical dilemmas and emotions in the practicum have a vital impact on their overall development as the most powerful determinants of future perceptions of what passes for acceptable behaviors and values in the practice of the profession and have a profound effect on students’ overall professional development in the future (Karnieli-Miller et al., 2010; Meijer et al., 2011).

The results of our study highlight ethical dilemmas faced by student teachers in the process of the practicum and illustrate the potential influences of their emotional experiences in responding to these dilemmas. Our intention of doing this research is to help teacher educators make explicit how student teachers experience ethical dilemmas during the practicum, and we hope that it will serve as a window for teacher educators and policy-makers on the hidden and informal curriculum perspectives beyond what student teachers may gain at universities through formal curricula.

As described in the previous section, we suggest a psychology course that focuses on emotional regulation strategies and establishing effective interpersonal relationships for student teachers. New approaches that better prepare them for successful interaction in complex teaching practices are also needed. For example, a three-step reflective approach could be introduced (reflection-before-action, reflection-on-action, and reflection-after-action) to help students make sense of their experiences and to be better prepared for the challenges that might occur to them in the teaching world. In order to empower students in the face of real dilemmas, we suggested using different real-life scenarios as case study examples. Therefore, cooperation between training institutions and the practicum schools should be promoted in terms of diversifying common areas of activity, especially to enhance dialog on issues and challenges student teachers might encounter during their practicum.

As a fast-developing country, China is currently experiencing educational and curricular reform, and teacher educators and student teachers in China stand squarely in the headlights of these changes. Most programs perceiving a “need” to begin training a new cadre of teachers who will possess the required skills and attitudes to meet the reformed new goal have begun to develop new courses and practices. Teacher educators have paid more attention to how traditional in-class educational programs and curricula are designed and delivered than they have on the roots of professionalism that develop in student teachers’ practicum experiences. This study aims, with the assistance of a psychological lens of emotionality, to offer a closer look at the student teachers’ experiences of ethical dilemmas during their practicum and how they influence students emotionally in order to offer an insightful view for policy-makers and the upcoming changes as an important reference. We hope it will help teacher educators to retain and develop new, high-quality teachers by garnering a better understanding of how ethical dilemmas and their emotional experiences influence student teachers’ learning and their further career choices.

## Data Availability Statement

The raw data supporting the conclusions of this article will be made available by the authors, without undue reservation.

## Ethics Statement

Ethical review and approval was not required for the study on human participants in accordance with the local legislation and institutional requirements. The patients/participants provided their written informed consent to participate in this study.

## Author Contributions

XW was responsible for the proposal of the research topic, research, structure, and manuscript writing. DHL was responsible for data, analysis, and revision. JYL and XW were responsible for data collection and collation. All authors approved the final version.

## Conflict of Interest

The authors declare that the research was conducted in the absence of any commercial or financial relationships that could be construed as a potential conflict of interest.

## Publisher’s Note

All claims expressed in this article are solely those of the authors and do not necessarily represent those of their affiliated organizations, or those of the publisher, the editors and the reviewers. Any product that may be evaluated in this article, or claim that may be made by its manufacturer, is not guaranteed or endorsed by the publisher.
